# It Takes a (Virtual) Village: Exploring the Role of a Career Community to Support Sensemaking As a Proactive Socialization Practice

**DOI:** 10.3389/fpsyg.2017.00097

**Published:** 2017-02-06

**Authors:** Darren Good, Kevin Cavanagh

**Affiliations:** ^1^Graziadio School of Business and Management, Pepperdine UniversityMalibu, CA, USA; ^2^Department of Organizational Behavior, Case Western Reserve UniversityCleveland, OH, USA

**Keywords:** career communities, sense-making, organizational entry, proactive socialization, growth, development

## Abstract

Scholars have long advocated for individuals to play a more proactive role during organizational entry rather than relying on institutionally led processes. The primary benefit being that the newcomer moves from passive recipient, dependent on the institution to highlight relevant information, to active agent with self-determined sources and methods to aid in adjustment. A virtual career community made up of 12 first year business faculty members was created to provide such a self-determined source of support during the transition from doctoral studies to full-time assistant professorship. After the entry period (1 academic year), the interactions in this community were used as data for a phenomenon driven research study. The results illustrate how a virtual career community could be used as a proactive socialization tool by encouraging sensemaking amongst first year faculty peers. The sensemaking process consists of perceived contrasts and tensions, followed by positive and negative self-disclosures, community feedback, and the experience of cognitive-behavioral shifts. The findings also expand the proactive use of external referents during organizational entry, which previously had only looked at friends and family members of the newcomer.

## Introduction

The transition period during organizational entry is critical for individuals (De Vos and Freese, 2011) compelling many organizations to provide formal orientation programs and onboarding activities to help socialize new employees. However, it is not possible for an organization to structure and deliver all the information and support for each individual need (Wanberg and Kammeyer-Mueller, 2000). As a result, scholars have increasingly advocated for individuals to play a more proactive role in such periods, rather than relying solely on institutionally led socialization processes (Ashford and Taylor, 1990; Miller and Jablin, 1991; Morrison, 1993; Chan and Schmitt, 2000; Wrzeniewski and Dutton, 2001; Gruman et al., 2006). From this perspective, the newcomer moves from passive recipient, dependent on the institution to highlight relevant information and sources to support them, to active agent with self-determined sources and methods to aid in adjustment (Morrison, 1993; Bauer and Green, 1994); which have been related to reductions in anxiety (Saks and Ashforth, 2002) and increases in role clarity and job satisfaction (Wanberg and Kammeyer-Mueller, 2000). Such methods commonly include engaging informally with other people in the organization to help make sense of emerging questions and concerns related to the new job (Ostroff and Kozlowski, 1992). In this regard, sensemaking can be viewed as the primary practice of proactive socialization.

Such proactive socialization is especially important to newcomers making the move from school to professional life. This tends to represent a particularly challenging and consequential career transition (Ashforth, 2001) layered with uncertainty, especially when it comes to adjusting to the affective and interpersonal aspects (Weidman and Stein, 2003). New faculty members often struggle during their first year to shift identities from full-time doctoral student to full-time faculty member. However, this conflict in identity creates a dilemma as new faculty may fear that seeking support from social resources inside their new departments may result in them being classified as incompetent (Miller and Jablin, 1991). Therefore, an alternative support structure to help make sense in this transition could be useful.

Incoming faculty members may find helpful support by joining career communities or “self-organizing, member defined social structures from which people draw career support,” (Parker, 2000, p. 30). These platforms assist individuals in taking on a more proactive role in their development. Such proactive socialization strategies seem to align with an emerging “career” perspective, which suggests that individuals should be empowered to guide their career development (Parker et al., 2004). While not applied explicitly to socialization strategies, such a career perspective can be seen playing out in a number of related arrangements such as peer-coaching (Parker et al., 2008), communities of practice (Brown and Duguid, 1991), and developmental networks (Higgins and Kram, 2001). Career communities often transcend the boundaries of any single organization (Parker, 2000) and can be developed by linking individuals from similar organizations or occupations (Brown and Duguid, 1991).

This paper analyzes a virtual career community that was established by a collection of first-year faculty members during their transition from graduate school. The career community was designed as a mechanism for social support through sharing similar experiences. At the conclusion of the academic year, the lead author decided to analyze the interactions to see what benefits, if any, occurred through the use of the career community. It is important to acknowledge that the current study was not originally designed as a research study. Rather, it was an exploration of the use of a virtual career community used by transitioning faculty members. The primary research question prior to analysis is how a virtual career community could serve as a resource in helping new faculty members make sense of their transition into full-time faculty life? This research question is pivotal for two reasons: (1) the transition period of organizational entry is critical for individuals (Wanous, 1992; Kammeyer-Mueller and Wanberg, 2003; Jokisaari and Nurmi, 2009; De Vos and Freese, 2011) and (2) the newcomer socialization strategies that are applied during transition (Hess, 1993) play an important role to long-term success in the organization.

## Literature review

### From doctoral studies to full-time faculty

The transition period from doctoral studies to life as a professor is critical for long-term career success (Ashforth, 2001). It is during such transition (Ibarra and Petriglieri, 2016) that first year faculty members engage most strongly in identity work regarding sense of self, career, and the interplay between the two (Van Maanen, 1998). The initial development of career and workplace identity is indicative of future career behavior (Frank, 1995; Bochner, 1997; Bauer et al., 2005), making the process important to both the newcomer and the academic institution.

Academics encounter specific inconsistencies between their perceived expectations and lived reality during organizational entry. While the task dimensions and subsequent knowledge and skills as faculty are relatively clear upon entry, the affective, and interpersonal aspects are often more difficult to manage and less understood (Weidman and Stein, 2003). For example, the first year of faculty life has been described as high in anxiety and psychological demand and low in experienced control (Karasek and Theorell, 1990; Sorcinelli, 1992). Further, Turner and Boice (1987) found that faculty newcomers expressed high levels of professional dissatisfaction because of unmet expectations in anticipation of an intellectually stimulating environment. Newcomers expect to join a community of scholars but in most cases are thrust into a high-pressure environment where they have to deal with increased workloads that hinder potential to create such a sense of community (Gappa et al., 2007). This leads to early career faculty reporting consistent feelings of isolation at work (Bowen, 1986), and a decrease in satisfaction ratings of colleague support over time (Olsen, 1993). The entry experience prompts new faculty to make sense of what is happening and who they will be in their career.

### How individuals engage in sensemaking as a practice of proactive socialization

Proactive socialization was originally defined as information-seeking behaviors newcomers exhibited in order acquire tactical and interpersonal knowledge of the organization and reduce uncertainty (Miller and Jablin, 1991). The primary focus of research in this area was on the “proactive” element, or the belief that individuals are in charge of their own learning and development. However, as organizations have become flatter in structure, scholars have shifted focus to socialization behaviors. These behaviors have evolved to include more than simply the acquisition of knowledge but instead whole-person behavioral self-management (Ashforth et al., 2007). Yet the primary approach to the inevitable uncertainty that any new job will create, is to acquire needed information and request feedback from others supporting why information seeking (i.e., sensemaking) is the most well-studied practice of proactive socialization (Saks and Ashforth, 1996).

Newcomers reduce uncertainty by seeking information and learning about the specific organizational landscape, the job tasks, and the role expectations (Chao et al., 1994; Chan and Schmitt, 2000). Newcomers proactively manage this uncertainty by engaging in an ongoing sensemaking process (De Vos and Freese, 2011). Sensemaking is defined as how individuals create meaning from the experiences they take in Weick (1995). As changes, surprises, and contrasts emerge from the transition, individuals must figure out how best to understand and respond (Louis, 1980). A common proactive socialization method for newcomers is to turn to others for information seeking and reality testing (Ostroff and Kozlowski, 1992; Morrison, 1993), essentially enlisting partners in the sensemaking and sensegiving process. This sensemaking can occur through social interaction with internal referent sources, such as fellow organizational members (Feldman, 1981) and external referent sources, such as friends and family (Settoon and Adkins, 1997).

Previous research has generally supported the notion that newcomers should enlist internal referents (e.g., supervisors and coworkers) for sensemaking (Falcione and Wilson, 1988) as these referents have shown to lead to positive outcomes in workplace adjustment (Morrison, 1993). This makes sense as internal referents can serve as credible sources of information for the newcomer based on experience with the particular role expectations and culture of the organization (Fisher, 1986). Internal referents possess the local-organizational interpretive schema from which to filter and ascribe meaning to newcomer queries (Feldman, 1981; Louis, 1983; Ostroff and Kozlowski, 1992; Morrison, 1993). However, a dilemma arises when newcomers fear that seeking support from internal referents may result in being classified as a nuisance or incompetent (Miller and Jablin, 1991). In fact, some research streams have shown that newcomers who seek-out internal support actually display lower self-reported ratings of performance, as they consider the need to request help as a sign of weaker performance and often receive mixed messages from internal referents which leads to more ambiguity and less clarity (Feldman, 1976; Graen, 1976; Jablin, 1987).

Most research that explores the proactive use of external referents only considers friends and family of the newcomer (Fisher et al., 1979; Miller and Jablin, 1991; Settoon and Adkins, 1997). This study looks to contribute to proactive socialization research by describing how an external peer group, through the use of a virtual career community, may be a valuable source of sensemaking during organizational entry.

### The rise and evolution of career communities

Career communities are “self-organizing, member defined social structures from which people draw career support,” (Parker, 2000, p. 30). Career communities are platforms to assist individuals in taking on a more proactive role in their career development. That is, individuals can better understand their own strengths and weaknesses and determine their own goals and efforts which may be needed to achieve them (Parker et al., 2004). The interaction in these communities provides individuals with a framework to make sense of their environment and allow individuals to interpret their workplace experiences in a space with peers who are well-suited to understanding the demands they are often faced with. In other words, these communities operate through a relational lens that assumes careers are the function of each individual's relationships and social processes instead of specific work-related functions such as task mastery (Hall, 1996). Through engagement in these communities, individuals are able to capture the “richness, uniqueness, and complexity” of their lives (Savickas, 1997, p. 11) which, when applied to a career framework, can assist with self-direction, and career decision-making (Hall, 2002). Furthermore, career communities also give individuals a relatively safe outlet to express themselves and the intensity of their emotions, which has been shown to impact sensemaking behavior (Solomon, 1997).

More often than not, the successes of these support communities are based on a fundamental level of inter-subjectivity between individuals participating within them. Dreier (1996) defines inter-subjectivity as shared consciousness among individuals who have similar cognitive and emotional processes. It is assumed that fellow first year business school faculty will be experiencing similar events and tensions, which helps in creating greater inter-subjectivity. It is through these communities enriched with inter-subjectivity that individuals can share their own experiences and grow from them, and make sense of others' experiences in a way that further contributes to their own development (Borradori, 1994).

Scholars and practitioners have advocated that peer support systems have proved beneficial because peers are able to provide psychosocial and vocational support to one another (Eby, 1997; Ensher et al., 2001). Peer coaching is an element of career communities which focuses on interpersonal development and support. Peer coaching is a process through which two or more colleagues work together to reflect on current practices; expand, refine, and build new skills. Previous research has shown that the best peer coaching occurs in situations when partners share equal status (Siegel, 2000), the focus is on personal and professional development (Seibert et al., 2001), and ample time is given for members to reflect and identify areas for improvement (Van Manen, 1977; Daudelin, 1996; Raelin, 2000). While external peer coaching and career communities have been utilized in support of career and leadership development (e.g., Kotlyar et al., 2015), we are not aware of their use as a proactive socialization tool.

Our discussion of career communities to this point have been on the benefits of participation in those communities in general and less on the changes of conducting such a community in a virtual setting. “Virtual” career communities are communities where relationships among members is had through an online medium such as a website or social media rather than in person. Ardichvili et al. (2003) describe two challenges often faced by virtual career communities: (1) developing trust among members of the community and (2) potential technological limitations among members. While the development of trust is important in any career community to allow for open sharing, it can be considerably harder in virtual communities if the participants are not familiar with one another. Technological limitations such as unfamiliarity with a website could also result in a lack of communication which would adversely impact the community. Extensions of these findings have been applied to virtual classrooms (Palloff and Pratt, 2007) and virtual consumer groups (Füller et al., 2006).

The current research examined whether an existing career community could serve as a proactive socialization tool upon entry into a first year faculty position. Specifically, we wanted to examine sensemaking practices that may have resulted from participation in the community as sensemaking is one of the primary outcomes of proactive socialization. Parker et al. (2004) outlines popular elements of career communities and this research meets the criteria for an occupational community (i.e., similar professional qualifications), support community (i.e., giving and receiving support through similar experiences) and virtual community (i.e., contact through means other than face-to-face interaction). This community was made up of 12 individuals who were making the transition from doctoral studies to first year of full time teaching and research. These participants interacted in a private website space—providing self-reflection and support to one another. Through grounded theory of the posted reflections and interactive responses, along with *post-hoc* interviews, we describe how external referents promoted and expanded sensemaking.

## Methods

### Phenomena driven research

This was a Phenomena Driven Research study (Schwarz and Stensaker, 2016) analyzing the experience of one of the current authors and 11 other first year faculty members. Therefore, participation must be viewed in two phases. The first phase was only related to participation in the career community. Participants were 12 first year faculty members making the transition from their doctoral programs. The members of the group were recruited using an informal snowball sample. A snowball sample (or chain sample) is a technique where existing subjects recruit future subjects. The first author, who created the career community, invited a colleague to participate who then asked another until 12 participants had agreed to participate. Thus, each of the members was previously acquainted with at least one other person in the group but in most cases each person was not acquainted with more than one other group member. The first author wanted to create the community as a means for participants to draw social support during the first year of faculty life. All of the members were trained at tier-1 research universities in the United States. Members were starting full-time professorial positions in teaching and research universities in the US, Canada, and the UK. The group included five men (one of which was an author on this paper) and seven women.

The purpose of the community was simply to provide support and the research question emerged only after the community experience had concluded. This second phase of participation included the obtained permission from community participants to use the written responses on the private website as potential data sources. All members of the community agreed to allow their responses to be used in the study. Both phases of the study are outlined in greater detail below.

### Phase one: career community procedure

Due to participants being geographically dispersed, a private website was created for participants to provide self-reflection and support to other participants. A set of prompts was created to promote reflection and initiate discussion topics (see Table 1). These prompts were used exclusively throughout the community phase. Participants were expected to write one reflection per week but encouraged to participate as respondents to others' written reflections as often as possible. Anytime a participant wanted to contribute to the website, there was a “New Post” button they would click that would bring up a forum-style response sheet. Once they had completed their reflection, they submitted the post to the website where other members of the community had the ability to respond. Thus, the website was constructed to appear in reverse chronological order so that the most recent reflections appeared first. In terms of participation, there were some participants who wrote more than once a week and others who wrote approximately every other week.

**Table 1:** **Weekly reflection prompts for participants to respond to**.

**Participant reflection prompts**
1: Describe an experience that occurred in your professional life during the past week that was particularly inspiring.
2: Describe an experience that occurred in your professional life during the past week that was particularly challenging.
3: Describe an experience that occurred in your professional life during the past week that was particularly surprising.

Participants wrote individual reflections and responded to others' reflections throughout one academic year (late August–May).

### Phase two: data analysis and formulation of research question

After the conclusion of phase one, two participants conducted phone interviews with the remainder of the participants to gather information pertaining to the virtual career community and its degree of usefulness throughout the year. At the conclusion of these interviews, the lead author wanted to further examine what themes would emerge from a more thorough examination of the websites data. A research colleague who was not a part of the community process was brought in to assist with the coding of the website. Each author independently coded the data by allocating open-codes to the online reflections and responses. The coding was done separately for two reasons: (1) to allow both researchers to form their own opinion on patterns and prevent *a priori* bias and (2) to allow each researcher to work at his own pace to prevent fatigue. Each author created a list of open-codes after which we met to discuss patterns in the reflections and responses. It was during this time that the researchers began to group codes into concept categories. The researchers then began the construction of a codebook to define each concept using examples from the virtual career community. At the completion of this process, 10 concepts were agreed upon and conceptually defined.

The researchers then used the aforementioned codebook to assist in another open coding process. This was done to make sure that all patterns were encased into a unique concept. After all the written reflections had been read and coded for a second time, the researchers again met to discuss the categories. The researchers went through the reflections together, line by line, and discussed distinctions throughout. When researchers disagreed, they discussed the discrepancy until one concept was agreed upon. These discrepancies often resulted from confusion with codebook terminology. As such, the researchers continued to update and refine the codebook. It was during this stage of analysis that the researchers also talked about the creation of new concepts. At the end of this process, the researchers agreed upon eight concepts.

The researchers then coded the reflections one final time and again met to compare codes. As with previous meetings, discrepancies were resolved through talking about disagreements until a final decision was made. Finally, the codebook and representative samplings of the data were distributed to two doctoral students. These students were able to code the text with 87 and 90% accuracy, respectively. The final codebook can be found in the Supplemental Materials section at the end of this article.

## Findings

In this section, we describe the findings from the aforementioned grounded theory analysis of the written reflections and responses from community members. Frequency data of coded themes and examples can be found in Table 2.

**Table 2:** **Frequency of coded themes with exemplary examples**.

	**Coded occurrences**	**Examples**
Contrasts and Tensions	193	Damn, it is taking me a long time to prepare for these classes, especially since I want to do a really good job. Instead of getting an early night's sleep on Wednesday, I had to take the campus shuttle home at 3 a.m., when I had finished the last, painfully minor details on my power point. I am currently in the throes of negotiating my time better, and the days seem much shorter than I remember them being just 2 months back. I might have to make this a goal of mine in a more explicit fashion.
**The disclosure cluster**	**138**	
Positive self-disclosure	59	I need to update my goals from the fall, but my positive report on that front is that my teaching scores zooooomed up from my first semester. I was hoping for a 0.3 increase. It was closer to 0.8! I am thrilled.
Negative self-disclosure	79	I definitely reached a breaking point about halfway through my Thursday night class this past week, where I just thought, “I don't want this semester to go on 1 more day longer than it has to. I have four more lectures and then presentations and finals, and I'm counting down the days. Please make this end.” I know I hit this point about halfway through each semester, but it kind of made me sad on several levels. I really like teaching, and have felt so positively up to now that it's kind of a bummer to have so much negative affect, especially during the class itself. This is my first semester teaching as a brand-new professor, and it seems so memorable and significant, and here it is almost over, and rather than savoring it, I'm just wishing it would end.
**The sensegiving cluster**	**131**	
Balancing feedback	77	These activities are not sounding like any real sources of connection for you. I wonder if it might be possible for you to look for a few activities that you can really get into (and maybe even bring other loved ones into) from a standpoint of authenticity and also be rewarded for, whether because it is high-visibility or because it fills a quota. Is that possible? I would love to read about you in a win-win like this.
Self-reinforcing feedback	54	Congrats on the publication! I can imagine that being a great relief for you as you start focusing on teaching right now. I also appreciate the humorous outlook are able to maintain during this process. Good luck getting into the system, and I am sure the students will understand if communication has to start on Day 1 of class.
Cognitive behavioral shift	103	I found myself using a different voice than in the conversations we used to have when I was a student. It essentially boiled down to me projecting an image of being in control, confident and professional, making, and owning my own decisions and interpretation of events, rather than a confused student constantly looking for guidance, not trusting my first instincts, being overly sensitive to cues from her about what was appropriate, etc. It seemed like she really noticed too! It was also reassuring to retell my transition narrative and remind myself that I am adjusting and handling everything really well, and not having crises, perceived, or actual, like I felt like I was having constantly as a doctoral student.

### Contrasts and tensions: the need for sensemaking

Organizational entry periods tend to be marked by contrasts and tensions that require sensemaking (Louis, 1980). As newcomers such contrasts seem more common as expectations are unclear and the analysis seemed to support this. Contrasts can emerge from perceived differences in organizational features between the doctoral granting institution and the current employer or even between how other people perceive the individuals. In one instance, a participant wrote that he/she was told by his/her department chair that he/she was “serious and intense” which surprised him/her because he/she had never known to be viewed that way. As the new role enfolds individuals often note the distinctions to previous ways of being. In the following quote, the individual discusses his/her models of research productivity:

I am focusing on the former sensation and trying to jump back into what I used to remember thinking was productivity in research. But frankly, I wonder if my models of productivity are even relevant after so much transitioning. I wonder how actual life will match up with the fantasies and then I try to tell myself to stop wondering and just start acting.

Tensions also seemed common for first year faculty members. Tensions often related to resource demands associated with managing research productivity, a full-time teaching load, and work/life balance. Tensions in the case of entry can be more significant since the newcomer does not have the requisite organizational experience or insider colleagues to process and enact the appropriate response. Therefore, the tension sparks the need for sensemaking. In the following quote the participant wrestles with the demands of grading:

When grading papers I go through waves of feeling toward the assignment. I am interested and engaged if I take my time and then give solid feedback. This takes way too much time and then I tend to speed up the process, and begin providing really generic feedback—which leads to a lack of engagement with papers. Then I decide to focus in again and read slowly and carefully—providing feedback. Then I realize that I will never finish in the time I have allotted myself and blow up the whole system giving in more superficial feedback then I provided before.

### Sensemaking through positive and negative self-disclosure

Community members engaged in further sensemaking by disclosing observations of perceived outcomes as they interact with the environment. Self-disclosure is a common sensemaking activity because disclosure, be it positive or negative, indicates the individual is open to testing the nature of their assumptions about reality. By self-disclosing what they think is important, they are inviting feedback from others that can support or disagree with their assumptions. Positive Self-Disclosure was indicated by a personal success or achievement. Though not exclusively, these are statements that a first year faculty member may not necessarily deliver to internal referents to avoid perceptions of being overly confident or may simply not have the internal relationships with which to do so. Statements made by participants often related to feelings of personal achievement, career congruence, and satisfying outcomes. In many instances, these disclosures served as a resolution to a previously mentioned tension:

I am fully caught up on all of my lecture-based work for the rest of the semester…the next 3.5 weeks. Almost a whole month ahead of me! I haven't felt this kind of relief ever as an instructor and quite honestly feel like I lack a frame to process it. I must admit to being thrilled and somewhat dumbfounded by the shift and free time.

In other cases, positive disclosures were related to satisfying outcomes that affected individual identity (e.g., becoming more confident) and organizational transition (e.g., making new friends/colleagues, playing a positive role in service to the school).

The sensemaking process was also furthered by offering Negative Self-Disclosure. This was indicated by self-effacing information, exposing potentially risky sentiments, or when any displeasure or disagreement with any current institutional policies, practices, or stakeholders (e.g., superiors, co-workers, and students) was made. These statements were often an admittance that could be construed as embarrassing or representative of some sort of “politically incorrect” belief, emotion, or behavior. As a newcomer these statements may be construed as being potentially risky to share with internal referents. These statements were often related to self-doubt prompted perhaps by being at an unfamiliar point in one's career. In the following quote a participant shares feelings of concern and struggle in regards to his/her teaching and research:

…about what causes the most panic—I think its a few things. First, it still feels daunting to find a way to weave together complicated ideas. I always want to frame/define EVERYTHING right up front but of course the choice to put one idea first means that another can't go first and I find those decisions difficult. Despite finishing the dissertation and successfully publishing a few things I still have complete doubt in the likelihood that I can actually complete something I start and I want it done before I start which is perhaps not the most useful mindset.

Another manifestation of Negative Self-Disclosure were complaints about the new organization and/or member of that organization. Such complaints about on-the-job annoyances may be less safe to share with internal referents for fear of offending someone or appearing ungrateful. Also institutional complaints can create a perception of being framed as a difficult individual (Settoon and Adkins, 1997). For example, take a classroom full of students that a new professor perceives as displaying “an incredible lack of engagement.” Offering this observation to other internal referents may lead to an association that the new faculty lacks teaching abilities. External referents (such as friends and family) may be able to conceptually wrap their head around the issue, but may fail to possess the necessary experience to fully empathize, make sense of, and assist with solving/supporting the problem. Furthermore, the community allowed for individuals to receive support if they felt their colleagues were not provided that type of environment:

A challenge I am putting to myself this year is to not get too bogged down in the negative energy some of my colleagues are displaying, and to develop professionally, keep my goals in mind, and dare I say enjoy the first year.

### Sensegiving: providing both balancing and self-reinforcing feedback

Community members oftentimes responded to other's initial sensemaking disclosures by providing various forms of feedback. Earlier, we discussed how self-disclosure is a common sensemaking activity because it indicates the individual is open to testing the nature of their assumptions about reality. Sensegiving offers the opportunity for other members of the community to interpret and respond to disclosures. Sensegiving is the “process of attempting to influence the sensemaking and meaning construction of others” (Gioia and Chittipeddi, 1991, p. 442). Sensegiving is inextricably linked to the sensemaking process, influencing how individuals recognize and interpret uncertain environments (Maitlis, 2005). The feedback served as a sensegiving mechanism and a way to continue and expand the sensemaking process for all community members (those who disclosed the information that is being responded to and those who witness the exchange as members of the community).

### Balancing feedback

Balancing feedback (Sterman, 2000) was meant to influence others toward a particular perception, emotion or behavior. It is meant to influence toward a shift in behavior or maintenance in present course of action, depending on the perception of the commenter. In one example, a participant made a comment about having trouble “translating the topic of Organizational Behavior into a language and delivery that really worked for the undergrad audience,” and reached out into the community for help with how to do that. Then another community member responded with the following:

This week I found great success with asking students the question, “So, Who Cares?” after we explore more theoretical links. “How can ___(insert concept)___/all of this/etc. help you to do a better job in your teams this semester and/or in the rest of your lives?” Maybe they think it makes me cool to seem like I am questioning my own class content, maybe it resonates with the disengaged and/or critical students, I don't know, but I see students shift in their chairs, sometimes smile, and—most excitingly—even raise their hands to respond to such prompts. What have you been trying to engage the students? How do you judge engagement within the class? Can we build anything extra into our systems here?

Student engagement and classroom tactics were a common topic that participants were interested in developing during the first year. Other issues that came up often during discussion tended to relate to the development of personal identity and confidence in ones' actions, usually referencing the shift from doctoral student to faculty member. Members of the community were able to offer support and often did so in the form of thought provoking questions designed to influence another to think on a more critical level:

What do you think it would take for you to become more stable and confident in your abilities? Is it more time, more affirmative data? I would be more than happy to ask you about it in a few weeks.

### Self-reinforcing feedback

Self-Reinforcing Feedback (Senge, 1990) was coded when statements were made in which a commenter expressed positive support to another's statement of accomplishment for an objective or task. The statement often was expressed to reinforce a specific behavior. For example, in response to a post about connecting with students, one commenter stated, “I'm really impressed by how much you engage with your students.” In response to a post about a journal publication, five different members posted some sort of congratulatory comment mentioning how important publications are “early in the process” and then continued to build on the momentum by referencing that the individual could now “focus more on your teaching.” The system of weekly reflections allowed for individuals to tell their story to the community over the course of an entire academic year, which enhanced self-reinforcing feedback as commenters built on previous postings:

Real writing—okay, now I'm really jealous! No doubt this productivity was enabled by your organization last week.

### Cognitive behavioral shifts: an outcome of the sensemaking process

Through ongoing disclosures and sensegiving among members of the community, coders noticed several elements of Cognitive, or Behavioral Shifting applicable to the workplace (Foldy et al., 2008). That is, individuals were likely able to expand understanding and adapt in significant ways as teachers, researchers, and organizational actors (based on the influence/sensegiving of others). For example, in a previously used example (from Balancing Feedback) a participant actually followed another's sensegiving by using the question “Who Cares” in his/her classroom in an effort to promote engagement inside of the classroom. This suggests that at times behaviors and tactics were altered as a result of participation. A separate participant responded in the following way to the sensegiving:

I really like your idea about using the question “Who Cares?” I'm so used to thinking about this from a research perspective, but it's valuable for the material we teach as well. It seems critical that students generate their own responses rather than having us feed it to them. I will definitely try this. I will definitely try to gauge engagement through the non-verbal cues you mentioned as well as participation.

This suggests that even the individuals who do not provide the original disclosure may be cognitively or behaviorally impacted by other's responses (i.e., sensegiving). These shifts in thinking and behavior that are an important result of having been a member in this community. This community encouraged individuals to ask questions, think conceptually, and formulate answers to the uncertainty they faced upon entry.

## Discussion

### Theoretical overview

This research sought to explore if a private virtual career community could be used as a proactive socialization tool amongst first year faculty peers to enhance sensemaking. This study is able to contribute to proactive socialization research by elucidating external “professional” referents as an additional source of sensemaking and support; expanding on prior socialization studies with friends and family serving as external referents (Settoon and Adkins, 1997). Therefore, we see this career community as one that provided participants the ability to reflect and make sense of their environment in a safe space they may not have had otherwise. This was in line with previous research on careers in academia, that explained that while the task dimensions as faculty are relatively clear upon entry, the affective, and interpersonal aspects are often more difficult to manage and less understood (Weidman and Stein, 2003). Themes found in the data suggest such intra and interpersonal aspects emerged, including individuals showing struggles with work identity, asking questions about how to react to coworkers and students, and how to build confidence.

We also felt the community itself helped to emphasize the “proactive” element scholars have advocated for in the socialization literature (Ashford and Taylor, 1990). Again “proactive” includes any activity an individual takes up to aid in adjustment that is beyond the formal process offered by the organization. Logging into a website to voluntarily share a reflection acts as the “proactive” element. These participants took surprises and contrasts (Louis, 1980) they faced in the workplace and then proactively tried to make sense of them with a community of others who may have had the ability to understand and provide support. In this regard, the findings expand the proactive use of external referents during organizational entry, which previously had only looked at friends and family members of the newcomer (Fisher et al., 1979; Miller and Jablin, 1991; Settoon and Adkins, 1997), to individuals who share occupational similarities beyond the boundary of the organization itself.

Perhaps most central to our question was the use of the virtual community for sensemaking during the transition period. Sensemaking is critical for reducing the uncertainty that is inevitable in organizational entry (Saks and Ashforth, 1997), especially during transitions from academic to professional life. The data from the community site suggested that participants were met with realities at work that did not match expectations, resulting in uncertainty and tension. This is consistent with prior research and suggests the need for sensemaking and relational support during such periods (Saks and Ashforth, 1997).

Participants seemed to use the community in a way that promoted sensemaking. Community members disclosed both positive and less flattering elements of their professional life during this time period. As described above, the hope was that having a “safe” space away from internal referents might promote greater sharing of experiences (i.e., sharing elements that they may otherwise not share with those internal to the organization). While we cannot conclude this to be the case; the data does illustrate a theme of self-disclosure. Such disclosure (if honest), is evidence of participants reflecting on and making sense of experience. It also provided other participants with relevant information to respond to (i.e., give feedback) and encouraged more sensemaking of their own experiences when such initial disclosures provoked additional meaning making. We hoped this would be the case as the participants, while at different institutions, were sharing the common experience of organizational and professional entry to a largely similar new job/role. For example, the following quote taken from the follow-up interviews illustrates how mutuality and sharing of these experiences in the community may open one up to wider sensemaking:

First of all…it has been so inspiring! From time to time I logged-in to read what's going on in other fellow…worlds. While reading…, I could reflect on my own situations. I was able to compare my own with others': For example, it was good to know that everyone felt stressful from teaching—I was not the only one! Moreover, I could learn how each…approached and solved issues from her/his classroom. For another example, I felt sorry to recognize that I was not as a good teacher as others. Lots of other colleagues tried to support and help their students; but I tried to avoid them in order for my own personal time.

Such comparisons may have been helpful in sparking additional sensemaking that is akin to “observing others” in the workplace during traditional organizational entry periods (Wanberg and Kammeyer-Mueller, 2000).

The data from the career community site also illustrates consistent feedback given to members of the community, often in response to self-disclosures. In addition to information seeking, feedback is the other critical process in sensemaking during organizational entry (Ashford and Black, 1996). Participants provided feedback that was positive, and seemed meant to encourage others to continue doing a given behavior. This type of feedback, or sensegiving, is important for the sensemaking process, as feedback is usually in short supply during entry (Ashford, 1986). The encouragement on the site, may have helped reduce uncertainties about quality and performance. Participants also provided constructive or corrective feedback that may have been meant to shift someone's behavior or framing of an event. It is common for new employees to misinterpret or remain highly uncertain about events (Ashford and Taylor, 1990). Again, this type of feedback (i.e., sensegiving) is critical for sensemaking during the transition as it provides information for participants to reduce discrepancy in uncertainty. While the data obviously does not tell us if the interpretations were helpful, aiding in improved socialization back at work, it does suggest a furthering of the sensemaking process that goes beyond simple one-way reflection.

Finally the data from the site suggests that at times participants read the disclosures/feedback and made a determination to alter something related to their behavior or thinking. While we do not have evidence of actual behavior change, data notes the intentions for such alterations. This appears to be at least superficial evidence of a complete sensemaking and sensegiving process between multiple parties (Sharma and Good, 2013).

Additionally in the follow-up interviews participants referenced that the community gave them “the opportunity to reflect” and to “make meaning of something I hadn't taken the time to make meaning of before.” Other community members referenced specific skills and abilities they felt the community allowed them to develop, such as “tactics, approaches, and inspiration” for the classroom. Also in the follow-up interviews participants made direct mention to the perceived helpfulness the site played in his/her newcomer adjustment during the career transition:

My whole transition has been more of an outside experiment because of this site. I am more in tune with the transition than I ever would have been otherwise. I am used to muscling through and reflecting later. Here, I felt more in tune with this being a transition period for me. I have more experience of being above the scene as opposed to just in it.

### Practical implications

Advances in technology have shifted the manner in which organizations function and the way in which people think about and perform their work (Coovert and Thompson, 2013). With so much change happening on the technological side of organizations, there is no limit to the *types* of support that people will be able to receive during times of *needed* support. The research contained within this report and the research being done in the field of career communities provides many practical implications that we hope may encourage Human Resources (HR) departments to use technology to create novel ways for newcomers to receive a mixture of internal and external support during transitional phases (Azeem and Yasmine, 2016). For example, HR departments could set up temporary networks within a larger organization to connect those in the organizational entry stage. While such an activity would seem to take a research stream based on user proactivity and seemingly turn it into an organizationally sanctioned activity, it would still encourage people to think in more proactive ways. Further, the communities that are set up could be deregulated from the organization itself and specifically focused on allowing the user to make meaning of the process.

HR departments may also establish career communities for employees during transitions other than organizational entry. Specific communities can be set up for employees undergoing promotions or entering a new type of job within the organization. These could range in specificity and purpose. For example, communities can be formed for employees who are making the leap from individual contributor to manager, a transition known to be difficult for many (Morris, 2001; Belker et al., 2012). HR personnel could form web-based community exchanges, in which different career community needs are posted, and then filled by employees from different organizations.

Academic governing bodies, like the Academy of Management, could establish similar networks for first year Ph.D. students at different schools to assist in their transition year as well. These networks could be focused on various interpersonal tasks such as finding a research advisor or teaching support. This would change the way networks are formed now—around one's research interest—and expand it to include new sources of support specifically related to support and sensemaking.

### Theoretical implications: future research directions

Future research may explore the characteristics of individuals who compose a given career community. In particular, individual resources (e.g., fluid intelligence, personality, and emotional intelligence) could play a role in sensemaking (Di Fabio and Saklofske, 2014). For example, individuals who have higher self-reported emotional intelligence perceive themselves as more able to deal with negative experiences (Grotberg, 1995) and to cope adaptively with adversity (Campbell-Sills and Stein, 2007). In the context of career communities, such individuals may engage differently. Perhaps they would not participate as frequently as they do not believe they encounter levels of difficulty that require sharing. Alternatively such a person may be more active in the sensegiving process given a perceived self-efficacy in dealing with conflict. Regardless there is a need to explore the individual differences of community members and potential relationships to behavior.

Future research may also study individuals who are involved in multiple career communities. This would allow researchers to expand on the arbitrary nature of labeling someone as “proactive.” The current literature doesn't distinguish levels of one's proactivity, instead only drawing distinction between user proactivity and institutionally led processes. Is there a tipping point in which an individual could be too proactive? Is there a dynamic between being proactive and relying on the institution that needs to be explored in terms of balance? Further, it would be interesting for future research to examine the structure of the stories individuals inside of these communities tell and how these stories may change over time. Do individuals become more confident with the social support they receive inside of the community or do they simply learn to use the community as a psychologically safe crutch to lean on?

### Limitations

There were some limitations of the current research that should be mentioned and addressed in order to assist scholars to develop future research around career communities. Some limitations include the lack of objective outcome measures related to socialization, a small sample size within one specific academic field, issues related to boundary conditions, and generalizability. The current research has offered one piece to an ongoing narrative and offers a call for a shift from more subjective experiences to more objective measures of these communities.

One key limitation to the research is that there were no objective outcomes related to socialization. Some of the themes suggest that individuals were becoming more socialized in their organizations but without more objective data this paper can only tell one part of a more complex narrative. This is not uncommon in career communities research as many who have explored these topics tend to do so around subjective career data which can present problems with validity and reliability (Stebbins, 1970). Future research should begin to explore outcome measures such as engagement and socialization measured both from the individual and others in the organization. This could be done by recording these measures at multiple times during the course of the transition period and comparison groups may include, for example, internal peer mentoring programs (Allen et al., 1999). Future research may also examine the effectiveness of these communities by taking measures of perceived satisfaction or sense of helpfulness at the conclusion of the community.

Another limitation of the current study was that this community was small (12 members) and focused only on first year business school faculty. Future studies may wish to expand the sample size and draw comparisons to other differing communities and non-community members. Perhaps simply encouraging regular journaling would accomplish a similar outcome and therefore could offer another comparison group. Adding onto this point, the community site was meant to highlight the distinction between internal vs. external referents. However from a systems perspective, the boundary could also be drawn around the academic discipline or even the larger field of academia. In such cases the internal vs. external referents also changes to include different sets of people. Therefore, future research may draw different boundaries, expanding the context to non-business school faculty and ultimately to members of non-academic settings who are entering a new job/career.

One final limitation is that community approaches often fall victim to the issue of generalizability in that particular voices inside the group have a possibility of dominating the conversation (Parent et al., 2000). Given that members in the current community were each able to write their own reflections every week, this limitation, while worth mentioning, seems to have been alleviated.

## Conclusion

Through qualitative analysis of a virtual career community of first year faculty members, we explored whether it provided sensemaking opportunities to its members. Proactive socialization has encouraged the use of internal referents to assist in making sense of and adjusting successfully during times of organizational entry. In this career community the use of peer external referents all went through similar experiences as each was a business school faculty newcomer. The members in this community appeared to engage in additional sensemaking as a part of participating. Such a use of external referents may present another tool for organizations to consider in supporting newcomers. Additionally, joining such a community may be another valid proactive socialization approach to expand sensemaking and improve adjustment.

## Ethics statement

Institutional Review Board at Christopher Newport University. Participants signed a consent form agreeing to use their online reflection posts and engage in an interview after the experience. Any post that was used in the paper was first shown to the participant who wrote it or said it to get their approval.

## Author contributions

DG: data collection, lit review, analysis, and write up. KC: lit review, analysis and write up.

### Conflict of interest statement

The authors declare that the research was conducted in the absence of any commercial or financial relationships that could be construed as a potential conflict of interest.
