# Supplementary material for: It Takes a (Virtual) Village: Exploring the Role of a Career Community to Support Sensemaking As a Proactive Socialization Practice
Source: Front Psychol. 2017 Feb 6;8:97. doi: 10.3389/fpsyg.2017.00097 (PMC5292408; doi:10.3389/fpsyg.2017.00097)
Supplement: Supplementary file 1 [file DataSheet1.docx]

**Appendix – The Codebook**

1. **Surprise/Contrast: SC** represents a statement or series of statements noting a subjective difference between expectations and perceived occurrences. It tends to be unknowable until the experience takes place providing new data for comparison/contrast. It is indicated when a person:
   1. Mentions a difference in how processes impact them (e.g. I thought I would feel scared to teach for the first time, but it was actually empowering)
   2. Suggests amazement, surprise, shock, astonishment, notice of coincidence etc.
   3. Noticing a difference between expectations and realities (e.g. a student who seemed disengaged turns out to be very engaged in subsequent interactions)
2. **Positive Self-Disclosure: PSD** represents a statement or series of statements suggesting an area of personal success or achievement. Most notably marked by sharing feelings of satisfaction or excitement usually connected with doing work in accordance with the way it is intended to be done. These are statements that a first year faculty member may not necessarily deliver to other new coworkers to avoid perceptions of overconfidence. It is indicated when a person:
   1. Shares a professional achievement and its merits
   2. Shares a professional outcome that is congruent with his/her aims as a scholar/teacher (e.g. helping a student secure an internship)
   3. Expressing satisfaction or excitement about an outcome (e.g. Getting a paper accepted to a journal or developmental opportunity)
3. **Negative Self-Disclosure: NSD** represents a statement or series of statements providing self-effacing information or exposing potentially risky sentiments. It may be an admittance that is embarrassing or representative of some sort of “politically incorrect” belief, emotion or behavior for a 1^st^ year faculty member in an academic setting. It is indicated when a person:
   1. Admits to a lack of personal focus, drive or quality of time investment
   2. Admits to feelings of inadequacy or inability (e.g. feeling like teaching is not as strong as other professors)
   3. Admits to behavior that would be deemed less than ideal for someone in his/her position (e.g. Completing tasks right before they are due, lack of preparation in teaching, or resenting having to perform a task required of the job.
4. **Sensegiving: S** represents a statement or series of statements that intend to influence others toward a particular perception, emotion or behavior. This can be influencing toward a change (**Balancing Feedback, BF)** or maintenance in present course of action (**Self-Reinforcing Feedback, SRF**). It most often occurs in responses to a blog post as opposed to an actual weekly post. It is indicated when a person:
   1. Expresses explicit agreement with someone’s choice of framing or behavior
   2. Challenges another to consider options (e.g. “Have you thought about…)
   3. Offers techniques, processes, best practices (e.g. One that that works for me is…)
5. **Cognitive Behavioral Shift:** **CBS** represents a statement or series of statements demonstrating a change or *desire* to change in one’s thinking, perception, emotion or behavior. The change/expansion may be about work, about oneself and identity, about others engaged in the work, institution etc. It is indicated when a person:
   1. Stated difference about feelings, thoughts, or behaviors which have changed from an earlier state (e.g. confused to inspired)
   2. States desire to notice new information, or experiment with new frames
   3. Highlighting new possibilities for thinking, perception, emotion or behavior (e.g. desiring to expand positive feeling one has for TAs to one’s students)
   4. Statements of actual shifts taking place in how one does approaches a situation
   5. Open to future possibilities or doing something one may not have done before (e.g. "I'd love to learn more about that" or "That Is something I will work on"
